# The Influence of Movement Initiation Deficits on the Quantification of Retention in Parkinson’s Disease

**DOI:** 10.3389/fnhum.2012.00226

**Published:** 2012-08-01

**Authors:** Lisa K. Pendt, Heiko Maurer, Hermann Müller

**Affiliations:** ^1^Department of Psychology and Sport Science, Justus-Liebig-UniversityGiessen, Germany

**Keywords:** Parkinson’s disease, motor learning, retention, initiation deficits, throwing movement

## Abstract

In patients with an impaired motor system, like Parkinson’s disease (PD), deficits in motor learning are expected and results of various studies seem to confirm these expectations. However, most studies in this regard are behaviorally based and quantify learning by performance changes between at least two points in time, e.g., baseline and retention. But, performance in a retention test is also dependent on other factors than learning. Especially in patients, the functional capacity of the control system might be altered unspecific to a certain task and learning episode. The aim of the study is to test whether characteristic temporal deficits exist in PD patients that affect retention performance. We tested the confounding effects of typical PD motor control deficits, here movement initiation deficits, on retention performance in the motor learning process. 12 PD patients and 16 healthy control participants practiced a virtual throwing task over 3 days with 24 h rest between sessions. Retention was tested comparing performance before rest with performance after rest. Movement initiation deficits were quantified by the timing of throwing release that should be affected by impairments in movement initiation. To scrutinize the influence of the initiation deficits on retention performance we gave participants a specific initiation intervention prior to practice on one of the three practice days. We found that only for the PD patients, post-rest performance as well as release timing was better with intervention as compared to without intervention. Their performance could be enhanced through a tuning of release initiation. Thus, we suggest that in PD patients, performance decline after rest that might be easily interpreted as learning deficits could rather result from disease-related deficiencies in motor control.

## Introduction

**Figure 1:**
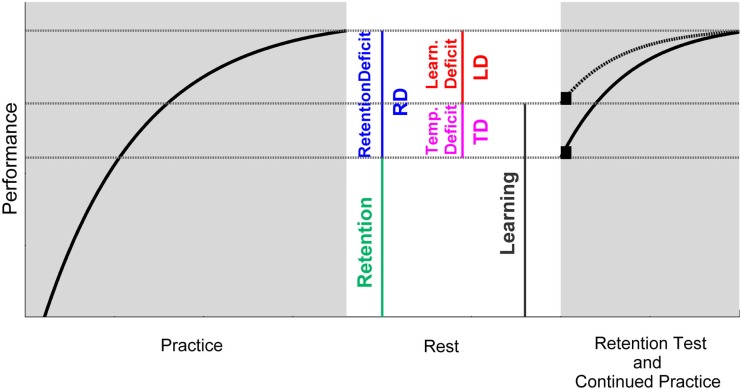
**Schematic illustration of the interrelations between pre-/post-rest performance, retention, temporary influences, and learning**. Retention is here defined as the performance remaining after a period of rest. The retention deficit (RD) is, hence, the difference between the performance at the end of practice (pre-rest) and after rest (post-rest). The RD can be composed of an actual learning deficit (LD) as well as negative influences arising from temporary factors (TD). Hence, these temporary deficits need to be taken into account in order to determine learning. If temporary deficits could be eliminated, retention performance would be higher as compared to when temporary deficits became effective (compare the black squares after rest). And this performance would represent what would have been learned. Since temporary effects vanish with further practice, performance curves of both scenarios merge after some practice trials (compare dashed and solid line after rest).

Motor learning is generally defined as a relatively permanent improvement in a motor skill as a result of practice (Schmidt and Lee, 2011). In behaviorally based studies, the improvement in a skill can be determined by a change (usually an increase) in motor performance over a practice phase (Figure 1). In order that such an improvement can be termed learning (in delineation from adaptation for example) it needs to be of relative permanence as the definition claims. This permanence is typically scrutinized by retention tests. Hence, the performance after the period of practice is compared to the performance after a period of non-practice, i.e., rest. Complete retention can be assumed when performance does not change over rest, while a decrease in performance indicates incomplete retention. In each case, the performance that has been retained over rest is taken as quantification of what has been learned. Accordingly, the difference between pre-rest and post-rest performance [the retention deficit (RD)] is interpreted as forgetting or, in other words, a learning deficit (LD). Thus, a manifest change in performance is ascribed to the latent underlying process of learning or forgetting, respectively. However, performance changes cannot only be brought about by learning and forgetting but also by other relatively task-unspecific temporary factors such as motivation, warm-up decrement (WUD), the current motor function, or fatigue. Imagine there is a negative influence of one or more temporary factors on post-rest performance as compared to pre-rest performance. If so, a performance loss over rest could not completely be ascribed to a LD. At least a portion of it has to be explained by a temporary deficit (TD), as we want to term it (Figure 1).

Especially in the context of motor disorders, the task-unspecific current functional capacity of the control system is one potential candidate for a TD. When comparing performance change between at least two points in time and if the current motor function at both times is not equivalent, a difference in performance cannot be fully ascribed to learning or LDs, which might lead to a faulty interpretation of the amount of learning. As a consequence, the condition of the functional capacity of the control system should be taken into account when investigating motor learning in populations with motor disorders. Parkinson’s disease (PD), for instance, is a disease where motor function is impaired through degenerative processes in the basal ganglia (BG). In addition, several lines of research provided evidence that BG are also involved in motor learning (Graybiel, 1995, 2005; Rauch et al., 1997; Doyon et al., 2002; Brasted and Wise, 2004; Fujii and Graybiel, 2005; Pasupathy and Miller, 2005; Nambu, 2008). With respect to the therapeutic context in PD, it is of great relevance to have insights in the ability of patients to learn motor tasks. Hence, a considerable number of studies has tried to answer the question whether and how strong PD patients are impaired in learning motor skills. It has been shown that patients with PD can improve in motor tasks when they are medicated and in a mild or moderate stage of disease. When compared to healthy aged matched controls, however, most studies find differences in improvements, as indicated, for instance, by flatter improvement curves in rotary pursuit tasks (Harrington et al., 1990) and serial reaction time tasks (Jackson et al., 1995), less successful achievement of a 90° relative phase pattern in a bimanual coordination task (Verschueren et al., 1997), and a less pronounced after-effect in visual and force field adaptation tasks (Krebs et al., 2001; Contreras-Vidal and Buch, 2003; Fernandez-Ruiz et al., 2003). Few studies find poorer retention as well, in bimanual coordination tasks (Verschueren et al., 1997; Mochizuki-Kawai et al., 2004) and in a movement scaling task (Smiley-Oyen et al., 2003). However, whether these differences can be interpreted as motor LDs in PD patients partly depends on the task-unspecific but disease-specific current motor function of the patients.

Even though it is discussed that motor learning deficiencies are uncorrelated to the general motor rating scores in PD (Heindel et al., 1989; Jordan and Sagar, 1994; Muslimovic et al., 2007), a few studies report influences of specific disease symptoms like bradykinesia (Harrington et al., 1990; Swinnen et al., 2000) and hypokinesia on changes in motor performance across practice (Soliveri et al., 1992; Pascual-Leone et al., 1993; Contreras-Vidal and Buch, 2003) or on performance in retention tests. For instance, in a study of Smiley-Oyen et al. (2003) PD patients showed poorer performance in a movement scaling task (underscaling) at the beginning of practice and at a 24-h-retention test which might be related to hypometria. The authors emphasize that the patients managed to make longer movements during the experimental session; hence they seemed to overcome hypometria with practice. Similarly, Swinnen et al. (2000) had PD patients practice a bimanual figure drawing task over 2 days and reported performance declines in the patient group at the beginning of new practice sessions that could be ascribed to bradykinesia and hypometria. Importantly, patients were also able to resolve these initial deficiencies in the course of each practice session. These findings emphasize the significance of the current functional capacity of the control system on motor learning. However, to our knowledge, the confounding effects of motor control deficits on motor learning have not been the focus of systematic research yet.

Pendt et al. (2011) have addressed this issue with respect to PD patient’s retention performance. They found that PD patients who practiced a virtual goal-oriented throwing task over 5 days showed similar performance improvements as healthy control subjects across days while their initial performance of each new practice session constantly decreased compared to the previous session. Patients needed about 25% of the practice trials of one session to reach the performance level that they had achieved before a 24-h break, but they managed to further exceed this level with continued practice. This initial performance decrease could be ascribed to problems in timing of the ball release. Relative to the timing before a practice break, PD patients released the ball, with which they had to hit a target, later after that break, leading them to miss the target and reducing their performance. The delayed release, however, was overcome in the course of further practice, resulting in more target hits and hence in a performance increase. With respect to the akinetic symptoms of PD, it is reasonable that the release delay was not an expression of poorer learning but rather related to the problems of PD patients to self-initiate a movement (Bloxham et al., 1984; Marsden, 1989). In other words, the performance decrease after rest was assumingly caused by a TD, i.e., by temporarily different conditions of the PD-specific initiation deficits at the end versus the beginning of a practice session.

This assumption would provide two important conclusions about motor learning in PD: First, performance declines after a practice break that might easily be interpreted as a LD could rather be caused by initial control deficits. Second, since the PD patients in that study overcame their problems in the course of a practice session, the movement initiation impairment might not be an irreversible deficit but can mitigate with practice.

To test these two assumptions we conducted an experiment in which participants practiced the throwing task from Pendt et al. (2011) over 3 days and received a deficit-specific but task-unspecific initiation intervention immediately at the beginning of one new practice session after a 24-h rest. The goal of the intervention was to attune to the release initiation (deficit-specific) in the ballistic throwing task without practicing the actual task (task-unspecific). Therefore, we used the same experimental apparatus but altered the task to minimize transfer from the intervention task to the experimental task. Hence, the intervention was expected to have a specific effect on timing and performance of the PD patients if the timing deficits of PD patients arose indeed from movement initiation deficits. However, since general effects of the intervention, like transfer or motivational effects, cannot be ruled out completely, a healthy control group was used to separate general from specific effects. Concretely, it was expected that the control group would show general effects of the intervention but no specific effects related to movement initiation, whereas in the PD group the specific effects should exceed general effects. Thus, in group comparison positive changes in timing and performance were expected to be higher in the patients compared to the control subjects.

## Materials and Methods

### Participants

Twelve patients with PD and 16 healthy subjects participated in the study. All subjects were informed about the purpose of the study and gave written informed consent. The PD patients were tested on medication and they all fulfilled the UK Brain Bank Criteria for the clinical diagnosis of PD. Demographic and clinical information of the participants is given in Table 1. Exclusion criteria for both groups were any other neurological disease or orthopedic issues and global cognitive deterioration as indicated by performance below 24 points on the German version of the Minimal Mental State Examination (MMSE; Kessler et al., 2000; originally Folstein et al., 1975). In addition, cognitive performance of PD patients was examined using the SCOPA-COG (Scales for Outcomes of PD–cognition) rating scale (Marinus et al., 2003). All participants had normal or corrected to normal vision. They were all right-handed and used their right hand for the task. Patients had either bilateral or right unilateral symptoms. The protocol was in accordance with the Declaration of Helsinki. It was approved by the Ethical Review Board of the Justus-Liebig University in Giessen.

**Table 1:** **Demographic and clinical characteristics of PD and control group**.

Variable	PD (*n* = 12)			CG (*n* = 16)		
	*M*	SD	Range	*M*	SD	Range
Age	63.5	11.5	45–78	64.6	9.6	47–80
Duration of PD (years)	6	2.9	2–12			
UPDRS motor score	32.4	7.8	18–46			
H&Y			2–4			
SCOPA-COG/MMST	26(median)		18–38			26–30
	28(median)		26–30	
Medication (mg): levodopa (*n* = 12)	517.2	266.3	187.5–900			
Carbidopa (*n* = 12)	126.3	67.3	47–225			
Entacapone (*n* = 4)	1050	300	600–1200			

### Task and apparatus

**Figure 2:**
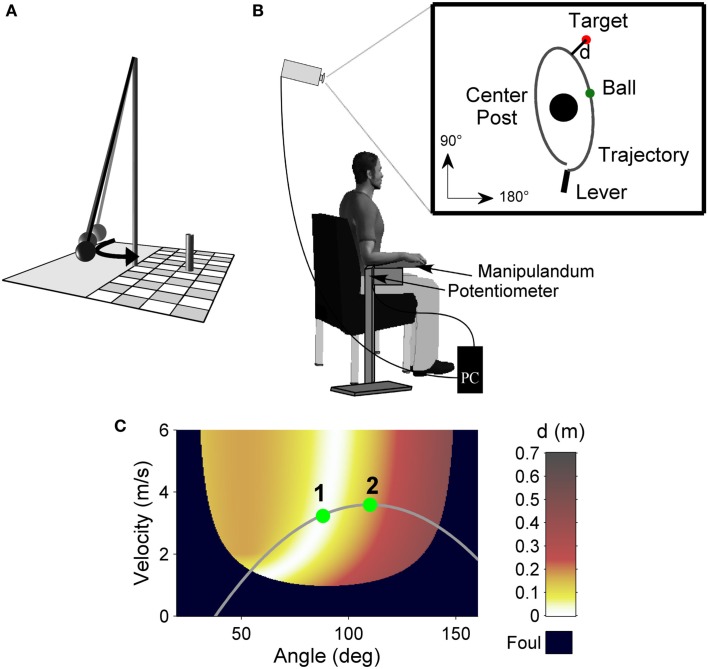
**Experimental task**. **(A)** Sketch of the real Skittles task. A ball is suspended on a string and swings around the center post, with the objective of knocking down the skittle at the opposite side. **(B)** Experimental set-up. Participants operate a lever to throw the virtual ball on the screen in front of them with the goal to hit the target located behind the center post. The angular displacement of the participant’s forearm is measured by a potentiometer and recorded by the computer. **(C)** Execution and result space of the Skittles task. For each combination of the execution variables (release angle and velocity) the color codes the result variable, the distance (*d*) of the resulting ball trajectory to the center of the target (error). White denotes the solution manifold with zero-error solutions. The gray curve represents a hypothetical throwing trajectory of the arm movement with two hypothetical release points and their respective timing. Release 2 occurs later on the throwing trajectory relative to release 1 and hence reaches lower performance.

The experimental task was a semi-virtual throwing task called Skittles that has previously been used (Müller and Sternad, 2004a,b; Cohen and Sternad, 2009; Pendt et al., 2011; Sternad et al., 2011). The idea of the task comes from a British pub game where a ball is suspended from a string attached to the tip of a vertical post. The player has to throw the ball around the post in order to knock down a target skittle on the other side (Figure 2A). The movements of the participants in the experimental task were real, whereas the ball flight was virtual. Participants saw the work space of the task in two dimensions from a bird’s eye view on the projection surface from which they sat approximately 2 m away (Figure 2B). The post in the center of the work space was represented by a circle of 0.25 m radius at position *x* = 0 m, *y* = 0 m. A circular target of 0.05 m radius was located to the right and above of the center post (*x* = 0.35 m, *y* = 1.0 m). The virtual arm was represented as a solid bar of 0.4 m length, fixed at one end. Sitting frontal to the projection screen, the participant rested his or her forearm on a metal arm (the manipulandum) with a plastic support padded with foam rubber. The manipulandum was fixed to a vertical support, which was adjusted to a comfortable height for each participant, and pivoted around an axle centered directly underneath the elbow joint. The elbow was fixed with a Velcro strap. Rotations of the arm were measured by a 5-turn potentiometer with a sampling rate of 1000 Hz. By touching an electrical switch at the free end of the metal arm, the ball was attached to the virtual arm on the projection. Upon releasing the contact, the electrical current was disrupted and this accounted as trigger for releasing the virtual ball. Participants first closed the switch with their index finger, then rotated the forearm in an outward horizontal motion, and simultaneously released the switch. The ball traversed on a trajectory initialized by the angle and velocity of the participant’s arm at the moment of release. Both the movements of the arm and the simulated trajectory of the ball were displayed on the screen in real time. The ball’s trajectory was determined by the simulated physics of the task and described an elliptic path around the pole. For details of the physical model see Müller and Sternad (2004b). The ball flight trajectory was not immediately intuitive to participants, and they had to learn the mapping between the real arm movements and the ball’s trajectories in the projected work space. Hence, the task was novel even for participants with extensive throwing experience. The center post between arm and target impeded trivial solutions, i.e., releasing with zero velocity. The minimal distance *d* of the ball flight trajectory to the center of the target was used to calculate a performance score, i.e., the score decreased linearly from 100 points for a perfect hit (*d* = 0.0 m) to zero for *d* ≥ 0.5 m (including center post hits). The relation between the execution variables and the result is displayed in the execution and result space in Figure 2C.

### Experimental design and procedure

The experimenter instructed participants to throw the ball in a counter-clockwise direction around the center post in order to hit the target. The movement direction was clockwise similar to performing a Frisbee backhand. After every 10 trials, a summed score was displayed on the screen. Participants were encouraged to keep their score as high as possible by achieving as many perfect hits as possible and by avoiding the center post.

**Figure 3:**
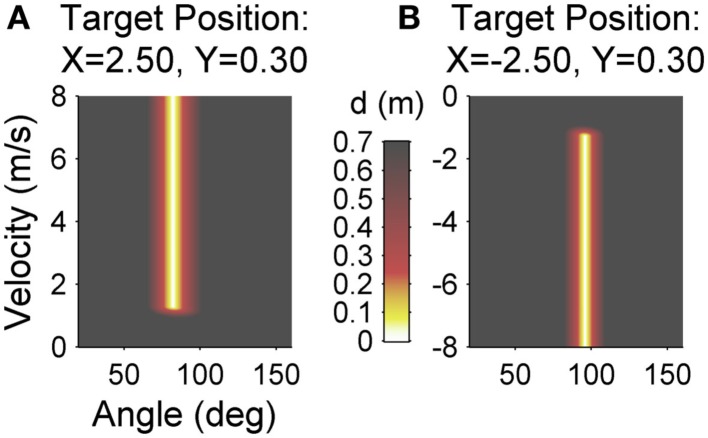
**Execution and result space of the intervention task for the left target (A) and the right target (B)**. For each combination of the execution variables (release angle and velocity) the color codes the result variable, the minimal distance (*d*) of the resulting ball trajectory to the center of the target (error). Red, yellow, and white areas denote target hits (*d* < 0.25).

Participants performed three experimental sessions on three subsequent days with 200 trials each. Sessions were scheduled within 1 h about the same time each day for individual participants such that rest between days was always about 24 h. The first day served as familiarization and acquisition of the task. Before the first practice session over 200 trials started, participants received task instructions. In addition, they tested apparatus and task for 30 trials with a different target position than during practice (*x* = −0.5 m, *y* = 0.0 m). Either on session two or three, participants then received an intervention to improve their release initiation prior to the Skittles practice. On the remaining session, there was no intervention prior to practice. The day with intervention was counterbalanced between subjects to control for sequence effects. The intervention consisted of a throwing task on the same apparatus. The task, however, was to hit two enlarged targets (radius = 0.25 m as opposed to 0.05 m) that appeared alternately on the right or left side of the screen (i.e., participants had to execute backhand as well as forehand movements). In addition, the ball did not describe an elliptical but a straight path tangential to the movement direction. As a consequence, the intervention task was less redundant and therewith had a stronger focus on release timing. The goal was to exercise release initiation without any transfer to the Skittles task. Figure 3 displays the execution and result spaces for both targets of the invention task for comparison to Skittles. The only feedback in the intervention task was whether the targets were hit or not. Subjects did not receive a score. In the study of Pendt et al. (2011), patients overcame their initial timing deficits after about 50 trials of practice. Hence, we chose 60 trials for the intervention task (30 trials on each target).

### Statistical analysis

With reference to Figure 1, we term the total performance change over rest as RD. The difference between a specifically quantified temporary factor related to movement initiation before and after rest represents the TD. On an abstract level, the subtraction of the TD value from the RD value would represent the amount of a LD. Since TD and RD differ with respect to their units, this subtraction has to be indirectly assessed by experimental manipulations. This was done by determining the different contributions of release timing (which should be indicative of release initiation) to RD for patients and control participants. This allowed deciding whether a reduction in performance after rest would result from a LD or the Parkinson specific initiation deficit.

For a first descriptive overview, performance score was averaged over blocks of 50 trials and changes in performance over practice blocks were analyzed with a 2 (group) × 12 (block) ANOVA with repeated measures. RD was quantified as follows: Mean performance of the last block prior to rest was subtracted from the first five series (10 trials each) after rest for each subject and intervention condition (intervention; no intervention). This was done to get a reliable reference of performance before rest and a sensitive measure of performance change after rest. The resulting variables were termed RD-Int_1…5_ for series 1 up to 5 with intervention and RD-noInt_1…5_ for series 1 up to 5 without intervention. Negative values in these variables indicate performance decrease, i.e., poor retention, after the practice break and vice versa. Thus, we tested whether the first post-rest series without intervention were less than zero (RD-noInt_1…5_ < 0), as would have been expected by the results of the study of Pendt et al. (2011).

To quantify the TD factor release timing we used a previously described method (Pendt et al., 2011) that determines the release points of different throws relative to each other since there is no common temporal reference for different throwing movements. In order to be successful, throwers have to first choose a throwing trajectory that intersects the solution manifold of the execution and result space (white area in Figure 2C). Second, they need to adequately time their ball release at that intersection such that the ball hits the target. It is assumed that participants produce adequate throwing trajectories once they have explored the task and created an internal model of it. However, release timing can still cause severe performance variations. Due to the short time window for release, even subtle changes in neuronal processing (e.g., initiation deficits) can have essential consequences on the result. Accordingly, one can analyze variability of release timing to infer such changes in motor control. To assess release timing variability we used a numerical procedure that aligns the angle time profiles of different throwing trajectories and therewith shifts their respective release points in time (see Appendix). As a result, release timing of each trial is expressed as a value in milliseconds. Timing is positive when release is delayed relative to the other trials and negative when release is early. A change in timing over rest was analyzed as follows: 50 trials before and 50 trials after rest were passed to the algorithm, resulting in a timing value for each of these 100 trials. Thereafter, average timing of the last block of trials before rest was compared to the first five series after rest analogously to the analysis of the performance score (TD-Int_1…5_ and TD-noInt_1…5_). However because a positive value represents delayed timing, the subtraction was reversed, i.e., the five post-rest series were subtracted from the corresponding pre-rest block, resulting in negative values when timing degraded and in positive values when timing improved. To test whether release timing degraded after a practice break we tested, analogously to the RD-noInt_1…5_ variable, whether TD-noInt_1…5_ < 0.

We had the *a priori* formulated hypotheses that the initiation intervention would improve release timing and strike performance after rest to a larger amount in the PD group than in the control group. Hence, for the analysis of the intervention effect we transformed RD and TD in order to directionally test mean differences. To do so, we subtracted for each subject and each series (1–5) the pre-post differences with intervention from the pre-post differences without intervention. As a result, we received five differences (one for each series: ΔRD_1…5_, ΔTD_1…5_) per subject that should have a positive value if intervention was effective and vice versa. It was expected that the intervention effect was predominant in the first series after rest and would recede in the following series. Therefore, we tested with a one-tailed *t*-test whether the difference between intervention and no intervention in the first series after rest (ΔRD_1_, ΔTD_1_) was more positive in the patient group than in the control group. In addition, we used a 2 (group) × 5 (series) ANOVA with repeated measures to test for a recession of the intervention effect in the patient group with continued practice after rest.

All statistical analyses were carried out using SPSS version 17.0. The level of significance was set at *p* < 0.05. If not stated explicitly, normal distribution of the data could be assumed or the characteristics of the samples (e.g., size) allowed parametric testing nonetheless due to the robustness of the utilized tests against such a violation. If variance homogeneity was not given, the Greenhouse Geisser correction was applied for the ANOVAs and Levene correction for the *t*-tests to adjust the degrees of freedom and control for the violation.

## Results

First, we tested for sequence effects, i.e., whether the day when the intervention took place (day 2 or 3) had an influence on the effect of the intervention. Because we did not expect differences between groups in this regard, patients and control subjects were pooled and were solely grouped by the factor intervention day. A 2 (intervention day) × 5 (series) ANOVA with repeated measures for the dependent variable ΔRD revealed no significant interaction effect day × series [*F*(4, 100) = 1.2, *p *= 0.32, η^2^ = 0.05] nor a main effect day [*F*(1, 26) = 0.1, *p *= 0.74, η^2^ = 0.07]. No significant effects were found for the variable ΔTD either [day × series: *F*(2.5, 62.9) = 1.5, *p *= 0.23, η^2^ = 0.06; day: *F*(1, 26) = 0.05, *p *= 0.82, η^2^ = 0.002]. Thus, we did not find a sequence effect regarding intervention day and therefore pooled the data of the intervention days for all subsequent analysis.

### Result variable: Score

**Figure 4:**
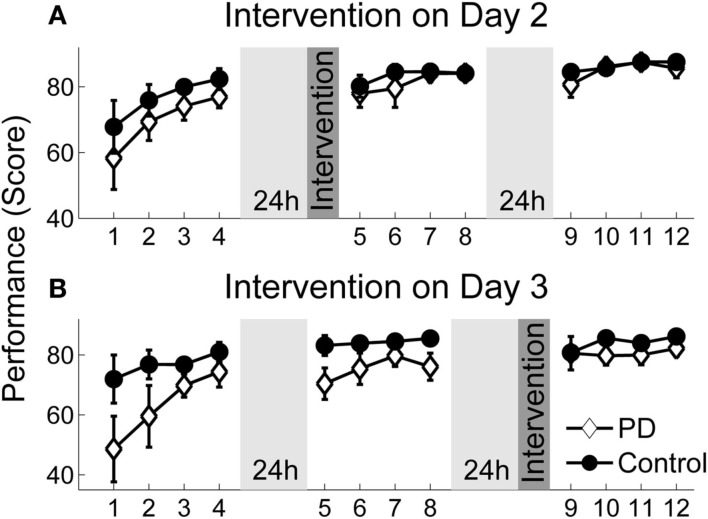
**Performance scores over practice**. Average performance scores over practice for patients and the control group assigned to their intervention groups. Each session consisted of 4 blocks (50 trials). There were 24 h breaks between sessions. **(A)** Performance change for the group that received intervention on day 2. **(B)** Performance change for the group that received intervention on day 3. Error bars denote the standard error of the mean.

Figure 4 displays average performance in score points over blocks of 50 trials for patients and control subjects separated by their corresponding intervention days (for better illustration). Before analyzing intervention effects, we tested how throwing performance generally developed with practice in patients and control subjects (independent of the intervention day). We could not observe normal distribution of the data and since the sample sizes of the patient and the control groups were unequal, performance changes were tested with the non-parametric marginal model (ANOVA type) of Brunner et al. (2002). The score increased across all blocks in both groups [block effect: *F*(6.4, ∞) = 2.5, *p *< 0.001]. Patients seemed to have a lower overall performance but the group effect was not significant [*F*(1, ∞) = 2.5, *p *= 0.12]. The block × group interaction was far from any significance either [*F*(6.4, ∞) = 0.8, *p *= 0.59], indicating a similar performance change over practice in both groups. Furthermore, both groups showed a performance decline in the first series after rest without intervention, as confirmed by a one-sample *t*-test for the variable RD-noInt_1_ across all participants (*t *= −2.6, df* *= 27, *p* < 0.01). However, when comparing post-rest performance between patients and the control group in Figure 4, one can notice a greater performance decline for the patient group in the blocks without intervention (block 9 Figure 4A and block 5 in Figure 4B) while post-rest performance with intervention (block 5 Figure 4A and block 9 in Figure 4B) was more similar in both groups.

**Figure 5:**
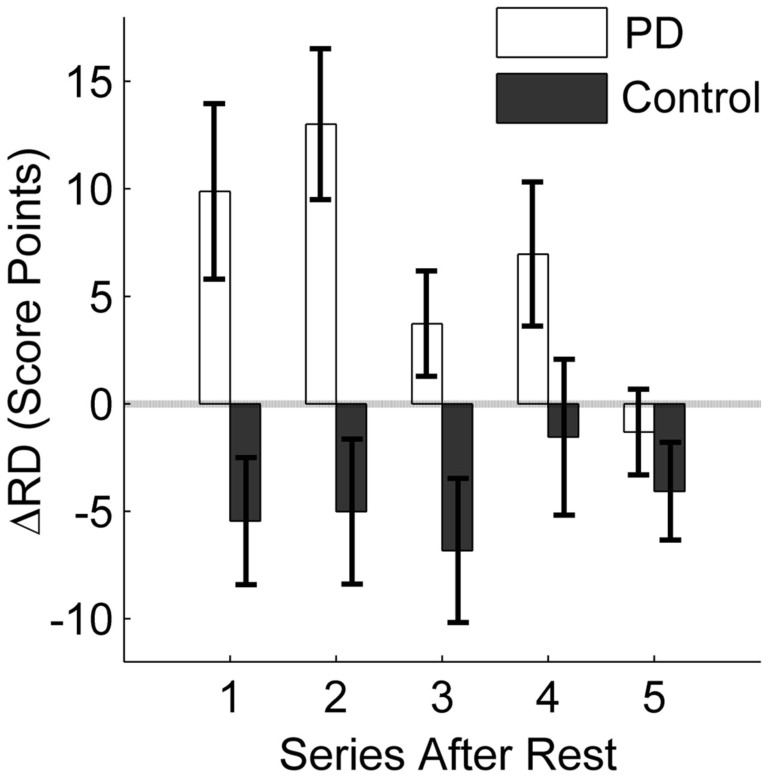
**Influence of initiation intervention on retention**. The difference between retention with intervention and without intervention is displayed. The average difference in score points for the first five series after rest (10 trials each) is displayed for the PD patients and the control group. Positive values indicate a positive effect of the intervention on retention; negative values result when the intervention leads to poorer retention as compared to no intervention. Error bars denote the standard error of the mean.

Figure 5 specifically displays the differences between groups regarding the intervention effect on the ΔRD_1…5_ variables. Performance of the patients did not reduce as much after rest with intervention as compared to the session without intervention. In contrast, the intervention had no positive effect on the performance of the control group. This was confirmed by a one-tailed *t*-test for independent samples for the first post-rest series ΔRD_1_ (*t *= 3.13, df = 26, *p *< 0.01). Thus, the initiation intervention helped only the PD patients to maintain their pre-rest performance. Furthermore, the group difference remained for the following post-rest series (ΔRD_1…5_), as revealed by the group effect of the 2 × 5 ANOVA with repeated measures [*F*(1, 26) = 11.98, *p *< 0.01, η^2^ = 0.32] even though the intervention effect in the patient group diminished with continued practice, indicated by a significant group × series interaction [*F*(4, 100) = 2.54, *p *< 0.05, η^2^ = 0.09].

### Timing of release: *Timeshift*

**Figure 6:**
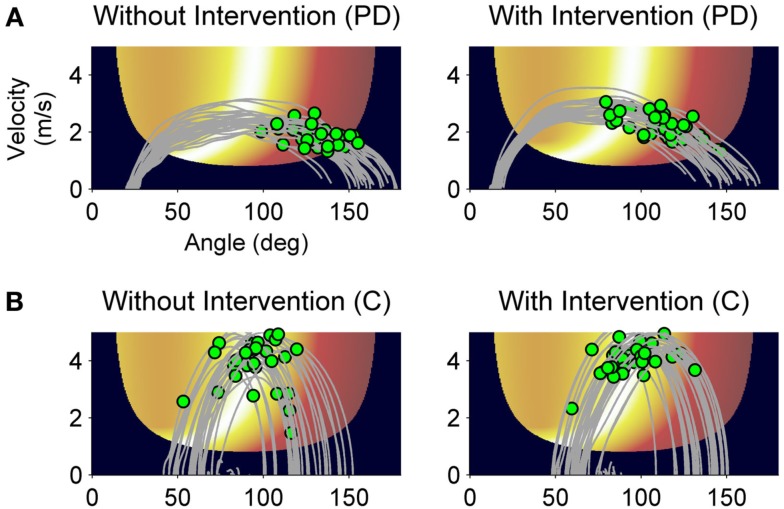
**Sample data of release control of a PD patient and a control subject**. Throwing trajectories (gray lines) and release points (green dots) after rest without intervention **(A)** and with intervention **(B)** plotted on the execution and result space. Movement direction is from left to right.

**Figure 7:**
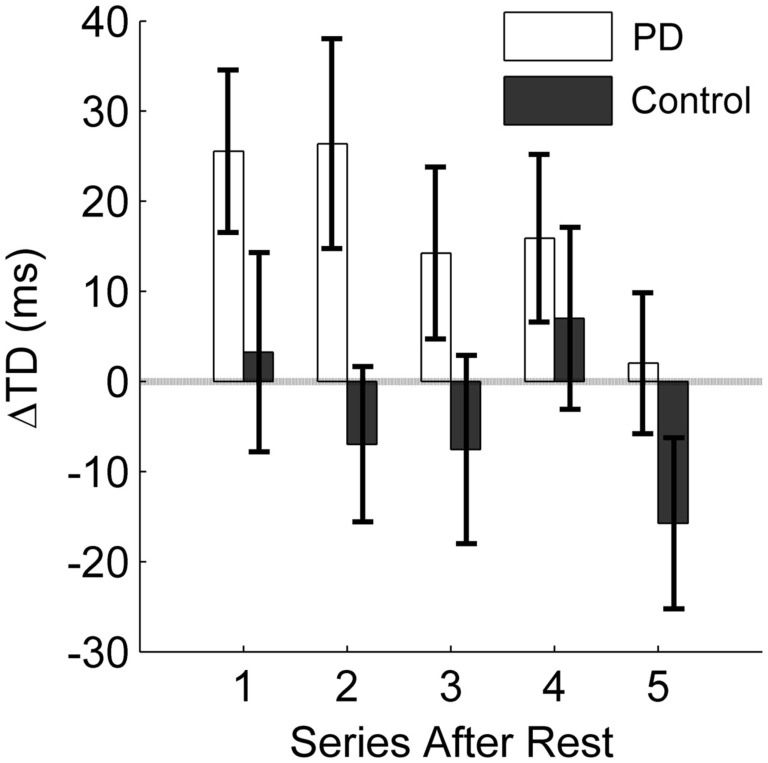
**Influence of initiation intervention on the temporary factor release timing**. The difference between a change in timing from pre-rest to post-rest with intervention and without intervention is displayed. The average difference in milliseconds for the first five series after rest (10 trials each) is displayed for the PD patients and the control group. Positive values indicate a positive effect of the intervention on timing; negative values result when the intervention leads to poorer timing as compared to no intervention. Error bars denote the standard error of the mean.

First, we analyzed the effect of the practice break on release timing without intervention similar to the RD-noInt_1_ variable. In both groups, release timing deteriorated after rest without intervention (*t* = −3.1, df* *= 27, *p* < 0.01). Although on average the decline was greater in the patients (*M* = 19.9 ms, SD = 18.5 ms) as compared to the control group (*M* = 10.0 ms, SD = 27.3 ms). Figures 6 and 7 show differences in the intervention effect regarding release timing between groups. As the sample data illustrate (Figure 6), release timing of the patients was better when initiation intervention took place prior to practice, whereas release timing of the control subjects did not change with the different conditions. The statistical comparison of a difference in the ΔTD variable confirmed these effects (Figure 7). The one-tailed *t*-test for independent samples confirmed higher positive ΔTD_1_ values for the patient group as compared to the control group (*t *= 1.8, df* *= 26, *p *< 0.05). This means that with initiation intervention timing after rest improved in the patient group while it had no effect on the control group. In contrast to the ΔRD_1…5_ variable, for ΔTD_1…5_ there was no significant group [*F*(1, 26) = 2.1, *p *= 0.15, η^2^ = 0.07] or interaction effect [*F*(2.0, 51) = 1.4, *p *= 0.25, η^2^ = 0.05] in the ANOVA, only a significant effect for series [*F*(2.0, 51) = 4.0, *p* < 0.05, η^2^ = 0.13]. However, when comparing Figures 4 and 6, a similar pattern can be observed. Namely, that the intervention effect diminished in the patient group within the first five post-rest series while changes in the control group were more unsystematic. This was confirmed by two *post hoc* ANOVAs with repeated measures for the ΔTD_1…5_ values for both groups separately [PD: *F*(1.6, 17) = 4.0, *p *< 0.05, η^2^ = 0.27; C: *F*(2.1,32) = 1.5, *p *= 0.23, η^2^ = 0.09].

## Discussion

Since the BG appear to be involved in motor learning processes, it is assumed that PD patients should show deficiencies in motor learning. Several studies confirm this assumption reporting differences between PD patients and healthy control participants in motor learning scenarios. However, there are hints that temporal reductions of PD-specific (but task-unspecific) motor symptoms might confound the quantification of motor learning, especially with respect to retention. Hence, the goal of this study was to examine these confounding effects of motor control deficits in PD on retention over rest.

In this study, we could show that, while practicing a virtual throwing task (Skittles) over 3 days, PD patients improved in strike performance on a similar level as a healthy control group. However, after a practice break of 24 h and when the new session started without any form of preparation, patient’s post-rest strike performance decreased. Typically, such a result would be interpreted as motor LD (RD). Yet, we found evidence that this performance decrease was rather caused by TD in motor control. Pendt et al. (2011) could already show that PD patients had extensive problems in release timing in the Skittles task after practice breaks. With the idea that these timing problems could be related to deficits in release initiation, we gave patients an initiation intervention after rest and immediately before they continued to practice Skittles. With this intervention, release timing and hence strike performance improved relative to when they did not receive an intervention. That means, PD patients were able to time their release better after rest with a deficit-specific but task-unspecific preparatory intervention compared to no intervention. In consequence, they hit the goal more often. The control group, on the other hand, did not benefit from the initiation intervention, neither in throwing performance nor in release timing. Post-rest performance of the control group did even decrease on average with the intervention (see Figures 4 and 5). Before we discuss this rather unexpected partial result, we want to draw a general conclusion first.

The intervention was designed to specifically affect release initiation. The result that this intervention helped the patients to enhance release timing and therewith strike performance after a practice break allows the conclusion that the performance declines observed after rest without intervention resulted from impaired release initiation. This interpretation is confirmed by the different effect of the intervention on the control group. Since it was not expected that healthy people had similar deficits in movement initiation as PD patients, the deficit-specific initiation intervention should not have had a positive effect on release timing in the control group.

However, there are some aspects to consider in respect of this conclusion. First, the effect of release timing on strike performance in Skittles is not linear. This becomes especially evident in the control group [compare the first post-rest series in ΔRD (Figure 5) and ΔTD (Figure 7)]. The negative effect of the initiation intervention on the score is not fully reflected in the effect on release timing. One explanation for this discrepancy lies in the redundant relation between execution variables (release timing, angle, and velocity) and the result variable in Skittles, i.e., the same strike performance can be achieved by an infinite set of solutions. Hence, this means that a later release does not necessarily yield a poorer performance and vice versa. However, this is only true for small changes in timing. Larger differences, as they occur in PD patients, do affect the performance as could already be shown in Pendt et al. (2011) and as patients’ results in this study demonstrate as well.

Second, strike performance and timing in the healthy control group also deteriorated after a practice break without intervention (although to a lesser degree as in the patients), likewise indicating a later release after the practice break as compared to the last trials before the break. But, here, the initiation intervention had no positive effect on timing after rest, i.e., it did not improve release timing in the healthy subjects, and it even negatively affected their strike performance. This negative effect of the intervention in the control group was rather unexpected. But, it can be explained with reference to a general negative transfer of the intervention task on the Skittles task. Since the intervention took place on the same apparatus as Skittles, it is plausible that different throwing strategies in the intervention task had interfered with the execution in the Skittles task. Given that the control group was representative of the general effects caused by the initiation intervention and because they showed a performance decrease with intervention, the general effect of the intervention can be assumed being negative. In contrast, the specific effect of the intervention in the PD group assumingly exceeded this general negative effect and hence, they showed a performance increase.

A question that consequently arises is: What is the cause for the poorer timing at the beginning of a new session in the control group considering that the initiation intervention did not help in their case? One explanation might be the influence of another temporary factor already mentioned in the introduction: a general WUD. The WUD is a short-lasting decline in performance after rest, typically observed in motor learning experiments over several practice sessions. Short-lasting in this sense is a relative term depending on the experimental task but usually lasting only a few trials following rest. It is possible that the observed timing problems of the healthy group in our throwing task might have been the effect of a WUD. At present, it is still not known what exactly provokes this decline but it is considered that the WUD is a “temporary loss of bodily adjustments or states” (Schmidt and Lee, 2011, pp. 477–478), i.e., the loss of some sort of calibration necessary to solve the task. Several studies have shown that an overcoming of the WUD seems to require a warm-up preparation that falls into the same movement class as the criterion task, i.e., that shares the same calibration characteristics with the criterion task (Nacson and Schmidt, 1971; Schmidt and Nacson, 1971; Wrisberg and Anshel, 1993). The initiation intervention did not help the healthy participants to overcome timing deficits at the beginning of a new practice session. Hence, one can speculate whether the intervention task and the Skittles task have distinct calibration patterns such that the intervention had led to a “wrong” calibration. However, at this point we cannot prove this. One would need to test different warm-up tasks to scrutinize whether a task with similar execution characteristics helped healthy subjects to improve in Skittles after rest to verify that their performance decrease had indeed been caused by a WUD.

Nevertheless, the patient and the control groups were differently influenced by the initiation intervention. This indicates that, in the patient group, the general effect of the initiation intervention was overruled by the disease-specific effect, namely the improvement of movement initiation. Thus, the main cause for the performance deterioration in the patients seemed indeed to arise from initiation deficits.

Another possibility to further scrutinize the different effects of the intervention in PD patients and healthy controls is the use of neurophysiological methods. One goal could be to scrutinize the effect of preparatory interventions like our initiation task on neuronal correlates of movement initiation.

Despite the questions still to be answered, the results already provide important remarks for the handling of motor learning studies as well as insights for therapeutic implementations in PD. First, the influence of motor function on performance and learning is a crucial variable that has to be considered when investigating motor learning *per se*, but specifically in populations with impaired motor control. The PD patients in this study showed decreased performance after practice breaks which, however, could be reduced by a simple intervention focusing on movement initiation. This indicates that the patients did not have problems learning the task, but rather their post-rest performance without prior intervention was degraded through a disease symptomatic initiation deficit. This is in accordance with other studies reporting intact long-term retention of a motor skill in corresponding patient populations (Agostino et al., 2004; Jessop et al., 2006; Smiley-Oyen et al., 2006; Pendt et al., 2011). Thus, taking into account the current functional capacity of performers reduces the risk of misinterpreting a lower performance gain as poorer learning. The consideration of such interrelations is essential with respect to adequate therapeutic implementations.

Furthermore, the constant improvement of the PD patient’s release initiation with practice demonstrates that at least mild to moderately impaired PD patients can enhance their motor symptoms through practice, which has been shown previously for other symptoms too (Behrman et al., 1996; Müller et al., 1997; Platz et al., 1998). Movement initiation deficits, in particular, are most frequently observed during gait in PD patients, but they are evident in all self-initiated movements (Edwards, 2002). Our results illustrate that for self-initiated ballistic arm movements like throwing, initiation deficits can be overcome with practice and, importantly, just as well with other preparatory tasks. As a consequence, therapeutic interventions should be designed in a way that patients have sufficient time to practice, especially to prevent frustration if a specific movement goal is not achieved immediately.

## Conflict of Interest Statement

The authors declare that the research was conducted in the absence of any commercial or financial relationships that could be construed as a potential conflict of interest.

## Figures and Tables

**Figure A1:**
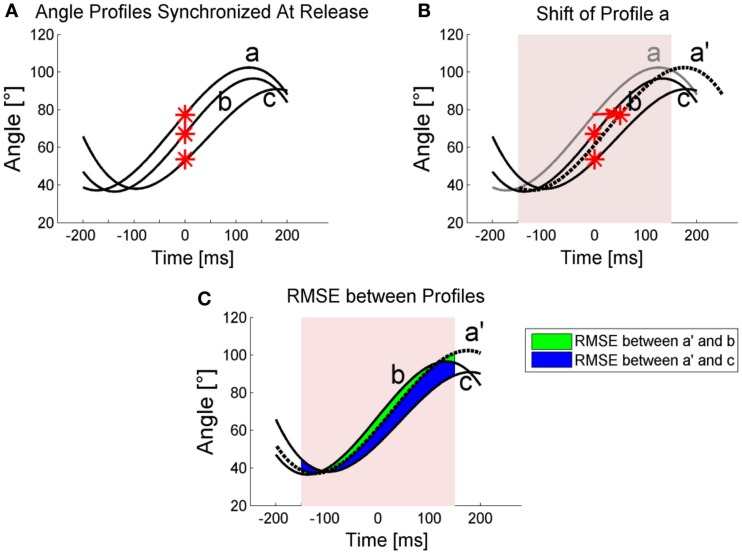
**Schematic illustration of the timeshift quantification**. **(A)** Angle profiles of 3 trials (a, b, c) are plotted with their release times (red stars) synchronized at time *t* = 0. **(B)** The alignment procedure shifts profile a by 50 ms to profile a′. For clarification a large shift value was used here. Note that in the procedure, the first shift value is 10 ms. The shift and the following RMSE calculation were done for 300 ms around release. **(C)** RMSE between trials. Note that for reasons of illustration, only areas between a′ and b as well as a′ and c for RMSE determination are displayed. RMSE between *b* and *c* was also calculated and all three RMSE determined total RMSE_t_.

**Figure A2:**
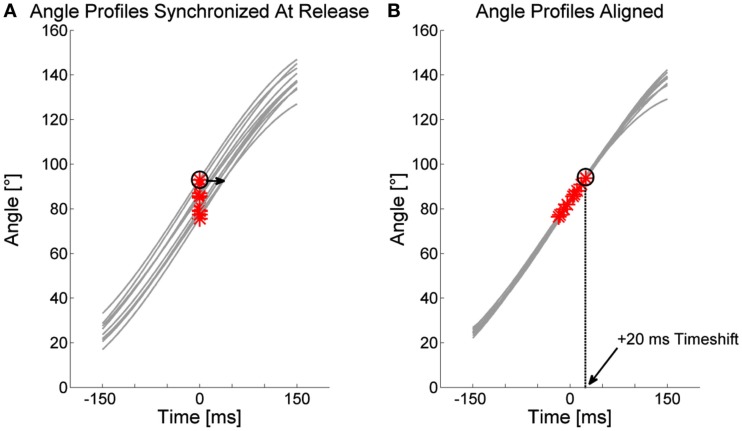
**Illustration of the timeshift measure**. **(A)** Angle profiles of 10 trials are plotted for a window of 300 ms around release and with their moments of release synchronized at time *t* = 0. The alignment procedure shifts angle profiles back and forth in time to reduce RMSE between the profiles. **(B)** Result of alignment optimization. Release points are positive or negative in the time dimension which equals the *timeshift* values. Encircled is one release point shifted by 20 ms by the procedure.

## Appendix

### Quantification of release timing using a numerical procedure

Assuming that after an exploration phase participants produce relatively stable trajectories over practice and only the release changes “over time,” release timing can be quantified using a numerical optimization procedure that aligns the angle time profiles of trials and allows assessing release timing variability.

We tested the assumption of stable trajectories within subjects by determining variances between angle time profiles of trials within subjects over the 20 practice blocks. The measure for variance was the root mean square error (RMSE) between angle profiles. As a result, RMSE reduced significantly after the third block and remained relatively constant until the end of practice. In addition, RMSE between trials within subjects was about 60% lower than a constellation of angle profiles randomly drawn from all subjects. Hence, subjects vary interindividually in their throwing manner, but intraindividually they are relatively stable.

The residual variance between angle profiles of different trials within subjects can be explained with variance in throwing trajectory, in particular changes in angle, or with variance in timing. We tested both alternatives and found a higher explanatory power of the timing hypothesis. Statistical tests secured this with a clear tendency. Thus, with reference to the rationale that, especially in PD, timing is more sensitive to control deficits than the generation of throwing trajectories, the *timeshift* analysis can be applied to quantify release timing.

In the following, the quantification procedure is explained by example of three sample trials (*n* = 3). Within the data collection procedure angle time profiles of trials were synchronized at the point of release (*t* = 0; Figure A1A). For a window of 300 ms around release, the angle profiles were shifted in time, with decremental steps starting from shift value *s*_start_ = 10 ms, to reduce pairwise distances. To minimize computational effort, coefficients of third order polynomials of the angle time profiles were determined using the function polyfit in MATLAB^®^, and the coefficients of each trial were shifted.

A custom-made algorithm in MATLAB^®^ was used for the following stepwise optimization procedure:

1. Shift trial a by the current time shift value *s_i_* (Figure A1B).
2. After each single shift, calculate the total root mean square error (RMSE_t_) between all polynomials (Figure A1C) and repeatedly shift trial a with shift value *s_i_* until RMSE_t_ does not reduce further. For the three sample trials, RMSE_t_ is computed as follows:
  (A1) $$\text{RMS}{\text{E}}_{\text{t}}=\text{RMS}{\text{E}}_{\text{a}\prime -\text{b}}+\text{RMS}{\text{E}}_{\text{a}\prime -\text{c}}+\text{RMS}{\text{E}}_{\text{b}-\text{c}}$$
  where RMSE_a′−b_ equals the RMSE between trials a′ and b, RMSE_a′−c_ is the RMSE between a′ and c, and RMSE_b−c_ represents the RMSE between b and c.
3. Repeat step 1 and 2 for all other trials. I.e., in our three trial example, trial b and after that c are shifted by shift value *s_i_* until RMSE_t_ does not reduce further.
4. Change the direction (positive versus negative) of the shift value:
  (A2) $$S_{i}=S_{i}*\left(-1\right)$$
5. Repeat steps 1–4 *n* times.
6. Change shift value direction and half the value size:
  (A3) $$S_{i}=\left(S_{i}*\left(-1\right)\right)∕2$$
7. Repeat steps 1–6 until the shift value is less than 0.1 ms.

This optimization procedure can move the release point of each trial backward or forward in time resulting in the *timeshift* measure in ms (Figure A2). *Timeshift* is positive when the release is delayed and negative when the release is early. The procedure can be applied to compare timing between individual trials as well as sets of trials. When two sets of trials are compared, trials of both sets are passed to the algorithm at once and angle time profiles are collectively aligned. After the procedure, timing information (*timeshift*) for all trials of set 1 are averaged and all trials of set 2 are averaged to be compared against each other.
